# Ovine leukocyte profiles do not associate with variation in the prion gene, but are breed dependent

**DOI:** 10.1111/age.12381

**Published:** 2015-12-20

**Authors:** Michelle R. Mousel, Stephen N. White, David R. Herndon, James O. Reynolds, Michael V. Gonzalez, Wendell C. Johnson, Massaro W. Ueti, J. Bret Taylor, Donald P. Knowles

**Affiliations:** ^1^ Animal Disease Research UnitDepartment of AgricultureAgricultural Research ServicePullmanWA99164USA; ^2^ Department of Veterinary Microbiology and PathologyWashington State UniversityPullmanWA99164USA; ^3^ Center for Reproductive BiologyWashington State UniversityPullmanWA99164USA; ^4^ Range Sheep Production Efficiency Research UnitDepartment of AgricultureAgricultural Research ServiceDuboisID83423USA

## Background

Lymphatic tissue is the accepted tissue type for live animal testing of scrapie, a prion disease in sheep,^1^, ^2^ for which genetic variation within the prion gene (*PRNP*) confers resistance.^3^ Circulating lymphocytes infected with PrP^Sc^ can cause scrapie in sheep.^4^, ^5^ In cattle, a lymphocyte profile has been found to be associated with a *PRNP* genotype^6^ and, if true in sheep, may have implications for live animal testing and disease progression. Therefore, our aim was to determine whether a sheep *PRNP* genotype was associated with leukocytes as measured by complete blood cell counts (CBC).

## Materials/Methods

Blood was collected from 589 ewes (Columbia, Polypay, Rambouillet, Suffolk) aged 2–6 years over 3 years (2011, 2013 and 2014) at the U.S. Sheep Experiment Station, Dubois, ID, USA, per USSES IACUC 10‐07 and WSU permit 3171. DNA was extracted, and CBC counts were recorded.^7^ The *PRNP* genotypes were identified with sequencing^8^ (data may be accessed at: http://www.animalgenome.org/repository/pub/USDA2015.0917/). A reduced statistical model, which included breed, genotype and age as fixed effects with CBC sampling year and sire as random effects, was analyzed with the Mixed procedure of sas 9.3 (SAS Institute). Genotypes with a lower than 1% frequency were removed from the analysis of that variant.

## Results/Conclusions

There were no associations (*P *>* *0.10) of any *PRNP* genotype with any leukocyte count except for the variant at nucleotide position 335 (amino acid 101) with monocytes (*P *<* *0.025) (Table 1). This difference is likely because only 10 heterozygotes were represented within these sheep and, if a Bonferroni correction is applied, there is no association. These results differ from cattle,^6^ but we did not differentiate lymphocytes into B cells, CD4+ and CD8+ T cells. Breed of sheep impacted (*P *<* *0.01) all leukocyte CBC measurements (Table S1). Generally, Rambouillet ewes had lower leukocyte counts than did ewes from other breeds and Suffolk had the greatest counts. Differences in leukocyte count due to sheep breed have been detected by others.^9^ Younger sheep had greater (*P *<* *0.01) WBC and lymphocyte counts than did older sheep (Table S2). Other counts did not differ (*P *>* *0.16) due to ewe age. We did not find an association of leukocyte counts, as determined by CBC, with the 10 *PRNP* genotypic variations detected here.

**Table 1:** Leukocyte means and standard errors per PRNP genotype/amino acid code

Nucleotide/amino acid	*n*	WBC^a^	Lymphocyte	Neutrophil	Monocyte	Eosinophil	Basophil
302/101							
AA/QQ	505	6828 ± 254	3351 ± 46	3085 ± 241	226 ± 7	167 ± 30	36 ± 4
AG/QR	80	6825 ± 309	3282 ± 98	3160 ± 275	234 ± 15	155 ± 34	32 ± 4
*P*‐value		0.987	0.475	0.622	0.628	0.563	0.269
335/112							
TT/MM	578	6844 ± 249	3340 ± 46	3105 ± 238	231 ± 7^a^	166 ± 29	36 ± 4
CT/MT	10	6557 ± 569	3537 ± 252	2931 ± 457	137 ± 40^b^	151 ± 61	26 ± 9
*P*‐value		0.592	0.444	0.671	0.025	0.789	0.217
407/136							
CC/AA	571	6827 ± 252	3343 ± 46	3101 ± 238	226 ± 7	165 ± 29	35 ± 4
CT/AV	18	6829 ± 436	3389 ± 179	2980 ± 361	242 ± 29	170 ± 47	38 ± 7
*P*‐value		0.995	0.803	0.675	0.598	0.911	0.544
421/141							
CC/LL	534	6837 ± 251	3356 ± 45	3095 ± 237	227 ± 7	167 ± 28	35 ± 4
CT/FL	53	6735 ± 327	3282 ± 111	3095 ± 285	223 ± 18	146 ± 36	37 ± 5
*P*‐value		0.653	0.518	0.997	0.819	0.380	0.611
428/143							
AA/HH	452	6769 ± 252	3324 ± 48	3052 ± 238	229 ± 7	168 ± 29	35 ± 4
AG/HR	121	7059 ± 283	3405 ± 79	3275 ± 257	230 ± 12	160 ± 32	36 ± 4
GG/RR	16	6923 ± 467	3501 ± 198	3126 ± 382	164 ± 32	155 ± 50	34 ± 7
*P*‐value		0.230	0.486	0.218	0.118	0.888	0.956
461/154							
AA/RR	556	6674 ± 372	3215 ± 140	3071 ± 317	223 ± 23	156 ± 41	29 ± 6
AG/RH	32	6834 ± 252	3351 ± 45	3095 ± 239	227 ± 7	166 ± 29	35 ± 4
*P*‐value		0.574	0.325	0.912	0.847	0.730	0.128
512/171							
AA/QQ	140	6866 ± 279	3248 ± 76	3243 ± 251	228 ± 12	159 ± 33	34 ± 4
AG/QR	283	6767 ± 258	3378 ± 56	3018 ± 238	226 ± 9	157 ± 32	34 ± 4
GG/QR	165	6918 ± 272	3379 ± 68	3124 ± 247	229 ± 11	185 ± 33	38 ± 4
*P*‐value		0.595	0.233	0.186	0.971	0.203	0.327
691/231							
AA/RR	473	6847 ± 254	3367 ± 46	3088 ± 238	228 ± 7	169 ± 30	36 ± 4
AC/RR	109	6747 ± 291	3217 ± 84	3146 ± 261	227 ± 13	149 ± 33	31 ± 4
CC/RR	7	6303 ± 643	3411 ± 290	2585 ± 509	193 ± 46	156 ± 68	25 ± 10
*P*‐value		0.585	0.189	0.466	0.759	0.539	0.100
710/237							
TT/LL	554	6833 ± 251	3348 ± 45	3091 ± 238	228 ± 7	167 ± 28	35 ± 4
CT/LP	35	6649 ± 368	3275 ± 138	3064 ± 314	208 ± 22	124 ± 40	33 ± 5
*P*‐value		0.509	0.592	0.887	0.371	0.139	0.600
711/237							
CC/LL	472	6850 ± 254	3366 ± 46	3091 ± 238	228 ± 7	169 ± 30	36 ± 4
CG/LL	110	6735 ± 291	3222 ± 83	3132 ± 261	226 ± 13	150 ± 33	31 ± 4
GG/LL	7	6301 ± 643	3412 ± 290	2582 ± 509	193 ± 46	156 ± 68	25 ± 10
*P*‐value		0.556	0.205	0.490	0.749	0.567	0.110

Different superscript letters indicate differences between genotype means at *P *<* *0.05 as tested by the Tukey–Kramer procedure in sas.

a Leukocyte numbers are count per microliter of blood.

## Supporting information


**Table S1.** Leukocyte means, standard errors, and *P*‐values by breed.

**Table S2.** Leukocyte means, standard errors, and *P*‐values by age in years.
