# Supplementary material for: Ovine leukocyte profiles do not associate with variation in the prion gene, but are breed dependent
Source: Anim Genet. 2015 Dec 20;47(1):136–7. doi: 10.1111/age.12381 (PMC4737273; doi:10.1111/age.12381)
Supplement: Supplementary file 1 — Table S1. Leukocyte means, standard errors, and P‐values by breed. [file AGE-47-136-s001.pdf]

Table S1 Leukocyte means, standard errors, and p-values by breed.

	Breed				
	Columbia	Polypay	Rambouillet	Suffolk	*P*-value
n	84	206	234	65	
WBC[1]	6824 ± 350[a]	6757 ± 338[a]	5916 ± 333[b]	7814 ± 376[c]	<0.01
Lymphocyte	3192 ± 122[a,b]	3405 ± 114[a]	2937 ± 114[b]	3930 ± 124[c]	<0.01
Neutrophil	3236 ± 302[a]	2866 ± 294[a,b]	2618 ± 291[b]	3440 ± 321[a]	<0.01
Monocyte	231 ± 19[a,b,c]	238 ± 18[a,b,c]	206 ± 17[b]	262 ± 19[c]	<0.01
Eosinophil	139 ± 38[a]	238 ± 37[b]	140 ± 36[a]	154 ± 41[a,b]	<0.01
Basophil	29 ± 5[a]	41 ± 5[b]	36 ± 5[a,b]	41 ± 5[a,b]	<0.01

[1] Leukocyte numbers are count per µl of blood.

[a,b,c] Different superscripts indicate differences between breed means at $P < 0.05$ as tested by the Tukey-Kramer procedure in SAS.
