# Supplementary material for: Ovine leukocyte profiles do not associate with variation in the prion gene, but are breed dependent
Source: Anim Genet. 2015 Dec 20;47(1):136–7. doi: 10.1111/age.12381 (PMC4737273; doi:10.1111/age.12381)
Supplement: Supplementary file 2 — Table S2. Leukocyte means, standard errors, and P‐values by age in years. [file AGE-47-136-s002.pdf]

Table S2 Leukocyte means, standard errors, and p-values by age in years.

	Age (years)					
	2	3	4	5	6	*P*-value
n	49	45	213	152	130	
WBC[1]	7738 ± 376[a]	7054 ± 381[a,b]	6654 ± 316[b]	6419 ± 324[b]	6273 ± 327[b]	<0.01
Lymphocyte	3904 ± 142[a]	3579 ± 145[a,b]	3226 ± 103[b,c,d]	3151 ± 108[d]	2968 ± 111[e]	<0.01
Neutrophil	3387 ± 319	3085 ± 322	2992 ± 279	2849 ± 284	2888 ± 286	0.163
Monocyte	262 ± 22	235 ± 23	225 ± 16	234 ± 17	216 ± 18	0.276
Eosinophil	162 ± 41	165 ± 41	187 ± 35	156 ± 36	169 ± 36	0.504
Basophil	44 ± 6	38 ± 6	33 ± 5	36 ± 5	34 ± 5	0.077

[1] Leukocyte numbers are count per µl of blood.

[a,b,c,d,e] Different superscripts indicate differences between year means at $P < 0.05$ as tested by the Tukey-Kramer procedure in SAS.
